# Gastric cancer and robotic therapy: Where we stand and where we’re headed

**DOI:** 10.12669/pjms.41.11.13105

**Published:** 2025-11

**Authors:** Wania Ashraf, Hamza Khan, Kainat Zahid, Javairia Shakil

**Affiliations:** 1 Wania Ashraf, Jinnah Sindh Medical University, Karachi, Pakistan; 2 Hamza Khan, Jinnah Sindh Medical University, Karachi, Pakistan; 3 Kainat Zahid, Jinnah Sindh Medical University, Karachi, Pakistan; 4 Javairia Shakil, Jinnah Sindh Medical University, Karachi, Pakistan

Gastric cancer (GC) remains a major challenge in the healthcare arena and has continuously emerged as one of the deadliest malignancies globally. Over the past years, its occurrence has been steadily increasing at an alarming rate especially in low-resource countries like Pakistan, where late diagnoses, screening schemes which are not sufficient, and the inaccessibility of surgical facilities are the causes of the issue. The significant difference between the outcomes of surgeries across countries also underlines the need to implement changes in the methodology and reduce the invasiveness, improve accuracy and speed up patient recovery.

Open gastrectomy with D2 lymphadenectomy traditionally has been considered a gold standard for curative care. Though this method has met with relative success, it is associated with greater invasiveness and found substantial blood loss, prolong hospital stays, and results in a large number of secondary surgical complications, particularly in older or comorbid patients. In the 1990s, Laparoscopic gastrectomy (LG) was proposed as an alternative minimally invasive technique and found to have many advantages as compared to open gastrectomy. However, it has the disadvantages of limited mobility of the instruments, two-dimensionality of laparoscopic imaging and challenges in accurately locating lymph nodes.^1^ With the continuous development and improvement of surgical techniques to treat GC, these shortcomings develop into highly evident ones, and researchers look into the potential of robotic-based surgery to provide the means of addressing them.^2^ It has been noted to enhance precision, surgical control and overall safety of the procedure, especially in complex cases.^3^

The scope of GC surgery over the last few years has expanded significantly and especially where careful tumor resection and the complete lymphadenectomy are necessary to achieve favourable oncological results. Despite a decreasing incidence of international GC, a steady rise in severe disease among individuals under the age of 50, highlights the potential need for more effective surgical interventions.^4^ The use of robotic gastrectomy (RG) is increasingly being used to replace LG overcoming its technical limitations. The da Vinci surgical system also allows greater dexterity, tremor-cancelling and scaled movements, whereby instruments are mounted on the wrist and thus allow a more articulated movement and more precise and controlled dissection within anatomically constrained spaces, a feature that becomes essential in D2 lymphadenectomy.^5^

Modern evidence suggests that RG is usually associated with lesser medians of outflow of blood as compared to LG. Moreover, previous reports suggest reduction in infection rates and anastomotic leakage, along with fewer complications compared to those of open surgery.^5^–^8^ A recent Phase-2 randomized trial supports this observation, showing that patients treated with RG spend less time in hospital (6.2 days vs. 7.4 days; mean difference 1.2 days) as a result of reduced tissue trauma and faster postoperative recovery.^7^ In addition, some data indicates reduced infection and anastomotic leakage rates, although these findings are not universally consistent across studies. Importantly, systematic reviews conclude that long-term superiority remains unproven, with recent umbrella reviews asserting that heterogeneity in existing research limits definitive conclusions.^9^ Remarkably, RG has shown oncological outcomes comparable to both laparoscopic and open procedures, so it is firmly gaining its place in GC treatment.

RG is a novel surgical technique that offers accuracy and low trauma, with insufficient utilization in low-and middle-income countries (LMICs). The increased operative time related to the robotic setup, which frequently takes place as a result of docking and instrument insertion is one of the drawbacks of robotic surgery.^10^ Marano et al. (2013) noted that RG does not result in more intraoperative blood loss compared to LG, (weighted mean difference=-35.5 95% CI: -66.98 to -4.09, P=0.03) in a random effect model (P<0.001, I2: 75%) but it takes (approximately 63.7 minutes) longer operative time. However, a recent study revealed that the complications, major morbidity, and lymph node retrieval were not significantly different using robotic and laparoscopic distal gastrectomy.^10^,^11^

A multicenter trial of 370 patients found that robotic gastrectomy is significantly more expensive than laparoscopic surgery ($13,432 vs $8,090, P < 0.001) but with similar outcomes.^12^ According to a public study carried out in Pakistan, the price of a robotic surgery was estimated at PKR 389,000 ($2,200), and excessive use of disposable and maintenance expenses were identified as the main disadvantages to the procedure.^13^ In an analysis of 1797, robotic surgery reported mechanical issues (around 2-3 %) of the procedures, with conversion rate to open surgery as low as 0.17%. Since the anomalies of instrument functioning and system failures are rare, the robotic system could be considered reliable in general.^14^ RG is otherwise uncommon in Pakistan due to high financial cost, absence of surgical training, institutional support and laparoscopic surgery continues to remain the most preferred surgery.^13^ By 2023, in Latin America, just a few publicly operated healthcare facilities, adopted robotic gastrectomy surgery, suggesting improvements beyond custom care but giving major regional disparities based on cost and training concerns, thus the necessity of country level data.^15^

However, many factors impede its usage in a global adoption including the prohibitive cost and the trained physicians and surgeons. Baral et al. (2022) emphasize that such research requires large-scale randomized controlled trials that would clarify the oncologic outcomes and cost-effectiveness of RG in the long-term application.^16^ In the same manner, Marano et al. (2021) report inconsistency in the existing evidence base, thus, identifying the need to conduct research of a higher quality.^9^ In order to increase the practice of the method, it is essential to organize developed training globally to enhance the level of robotic surgical skills especially among the low- and underprivileged nations. Some present information concerns the da Vinci® system, more recent construction hopes to bring costs down and make them more accessible, which includes Senhance® and Versius®. The inclusion of these systems could be critical to increase RG access in low-income settings.^17^

In addition, there is the need to transfer the technology and build capacity between well financed and underserved areas to popularize robotic surgery among the citizens. Robotic surgery must not remain a privilege that is enjoyed by fully well-resourced health systems. Although RG can be considered as an evidence-based treatment method with a few technical constraints, the effective practice of this approach should be based on high-quality evidence, training and inclusion which is outlined by Marano et al., Baral et al., and Manara et al.^9^,^16^,^18^ Surgeons, hospital administrators, and policy makers ought to invest in fair and evidence-based programs on robotic surgeries that should as well be encouraged. The innovation of access represents the GC surgery future, which implies that every corner of the world will be reached with lifesaving technology soon, safely and fairly.

**KEYWORDS:** Gastrectomy, Gastric cancer, Robotic Laparoscopic Low-And Middle-Income Countries.
